# Application of High-Density Electropulsing to Improve the Performance of Metallic Materials: Mechanisms, Microstructure and Properties

**DOI:** 10.3390/ma11020185

**Published:** 2018-01-24

**Authors:** Yinying Sheng, Youlu Hua, Xiaojian Wang, Xueyang Zhao, Lianxi Chen, Hanyu Zhou, James Wang, Christopher C. Berndt, Wei Li

**Affiliations:** 1 Institute of Advanced Wear & Corrosion Resistant and Functional Materials, Jinan University, Guangzhou 510632, China; iwrmsyy@163.com (Y.S.); huayoululaoda@163.com (Y.H.); Hc_sunboy@163.com (X.Z.); zhou_hanhany@163.com (H.Z.); 2 Reasearch & Development Center of Engineering Technology, Dongguan Eontec Co., Ltd., Dongguan 523000, China; 3 National Joint Engineering Center of High-Performance Wear-Resistant Metallic Materials, Guangzhou 510632, China; chenlianxigood@stu2014.jnu.edu.cn; 4 School of Engineering, Faculty of Science, Engineering and Technology, Swinburne University of Technology, Hawthorn, VIC 3122, Australia; jawang@swin.edu.au (J.W.); cberndt@swin.edu.au (C.C.B.)

**Keywords:** high-density electropulsing, recrystallization, solid phase transition, oriented microstructure or texture, resistivity of materials, dislocation behaviour, mechanical properties, crack healing

## Abstract

The technology of high-density electropulsing has been applied to increase the performance of metallic materials since the 1990s and has shown significant advantages over traditional heat treatment in many aspects. However, the microstructure changes in electropulsing treatment (EPT) metals and alloys have not been fully explored, and the effects vary significantly on different material. When high-density electrical pulses are applied to metals and alloys, the input of electric energy and thermal energy generally leads to structural rearrangements, such as dynamic recrystallization, dislocation movements and grain refinement. The enhanced mechanical properties of the metals and alloys after high-density electropulsing treatment are reflected by the significant improvement of elongation. As a result, this technology holds great promise in improving the deformation limit and repairing cracks and defects in the plastic processing of metals. This review summarizes the effect of high-density electropulsing treatment on microstructural properties and, thus, the enhancement in mechanical strength, hardness and corrosion performance of metallic materials. It is noteworthy that the change of some properties can be related to the structure state before EPT (quenched, annealed, deformed or others). The mechanisms for the microstructural evolution, grain refinement and formation of oriented microstructures of different metals and alloys are presented. Future research trends of high-density electrical pulse technology for specific metals and alloys are highlighted.

## 1. Introduction

High-density electropulsing treatment (EPT), also known as high density electric current pulse (ECP) treatment [1], is a new microstructure strengthen approach that applies a high density of electric current pulse to a bulk material in a short duration. As early as the 1990s, scientists and engineers have studied the effect of electric current pulse on the structures and properties of metallic materials by investigating ‘electromigration’ [2,3] and the ‘electro-plasticity effect’ [4] in metals. Subsequently, electric current pulses were applied to a variety of metals and alloys, including copper [5,6,7,8,9,10], titanium [11,12], magnesium alloys [13,14,15,16], aluminium alloys [17,18], tungsten [19], steels [20,21,22,23,24], shape memory alloys [25,26,27,28,29], amorphous crystals and metallic glass [30,31,32,33,34]. The influence of electropulsing on the properties and performance of metallic materials depends on their original microstructure, crystal orientation, crystallinity and the degree of deformation. As a result, different effects on the mechanical properties and performance of these materials have been observed. It has been reported that EPT of metals induced the evolution of the microstructure, such as grain refinement, the texture evolution and formation of oriented microstructures and the redistribution of inclusions [35]. Generally, small and uniform grains are beneficial since the material can deform uniformly under an external force, thereby enhancing the mechanical properties. In addition, the application of the pulse current improves the distribution of macro and micro defects and, in some instances, can reduce or eliminate these defects in many metals and alloys. Therefore, EPT has been used to heal microcracks in metallic materials [36,37,38]. EPT has been used for auxiliary turning operations to enhance the plastic deformation and cutting performance of the workpiece [39,40]. In addition, electropulsing could induce the redistribution of particles in liquid suspensions and helps clean molten materials from insulating oxides [41,42] and electrically neutral non-metallic inclusions [43]. By combining EPT with other material processing techniques, such as conventional heat treatment [44], high energy electron beam treatment [45] and ultrasonic shock treatment [46,47], enhanced material properties and performance could be achieved, including improved dynamic ductility of alloys due to the formation of ultra-fine grains microstructure and higher surface quality of alloys [48,49,50].

Up to now, there is still no unified recognition of the microscopic mechanisms during EPT. For instance, researchers have investigated: (i) the rapid temperature changes of heating and cooling during EPT [51]; (ii) the inherent characteristics of the current, such as current density and pulse width; (iii) the effect of additional free energy and current on the movement of electrons; and (iv) the induced thermal stress to different degrees. Recently, the multifactor theory has been developed, which includes coupling of thermal and athermal effects with associated [52], accumulation and annihilation effects of dislocations [53].

In this review, the progress of EPT on different metals and alloys is summarized. The theory of EPT and the influence of processing conditions on the properties of materials are discussed and further research directions in this field are prospected. 

## 2. Effects of EPT on Microstructure and Texture

**Figure 1:**
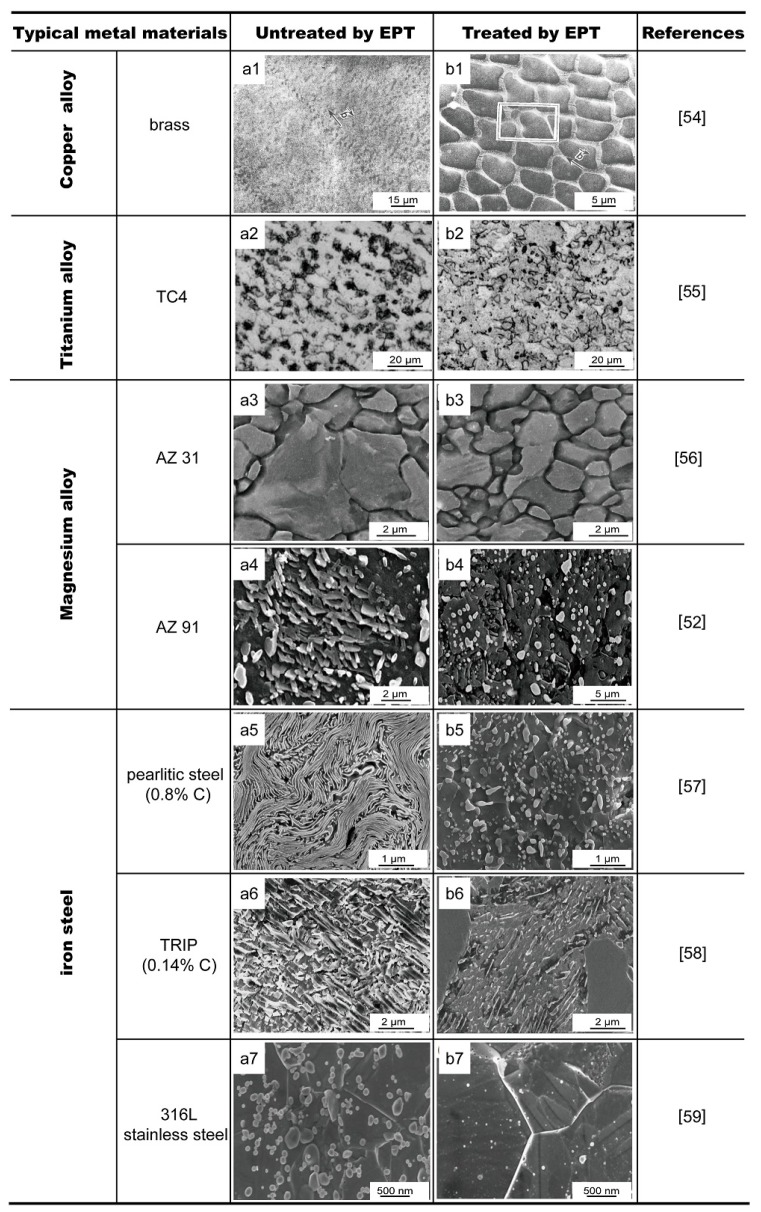
The microstructure of different alloys before treatment (**a1**–**a7**) and after electropulsing treatment (EPT) treatment (**b1**–**b7**).

EPT has been applied to many metals and alloys to achieve ultrafine-grained microstructure and nanostructures that arise from the solid state phase transitions and the process of recovery and recrystallization during their treatment. Figure 1 shows the variation in microstructures of copper alloy, aluminium alloy, magnesium alloy, iron and steel before and after EPT [52,54,55,56,57,58,59]. In addition, the pulse current could induce the texture evolution, resulting in the formation of oriented microstructure [1].

### 2.1. Recrystallization and Grains Refinement

EPT could increase the nucleation rate, reduce the temperature of dynamic recrystallization and promote grain boundary sliding. Conrad et al. [60] applied high density DC electropulses at a current density of 800 A∙mm^−2^, a frequency of 2 Hz and a duration of 90 μs to cold-drawn copper during annealing at 538 K. In comparison to conventional annealing, grain refinement was observed at a lower temperature, while grain growth was observed at a higher temperature (548 K). However, a continuous DC current (10 A∙mm^−2^) at the same heating rate did not reveal the same impact on recrystallization and recovery. It is believed that the pulse current affected the nucleation rate of equiaxed new grains. Additionally, the local thermal effect and the interaction of electronic defects may also be the reasons for the microstructure evolution of copper. Polygonal-recrystallized grains between 7.2 and 8.8 μm and elliptical-recrystallized grains between 5.3 and 8.0 μm were respectively observed in venation structures and resident slip bands of fatigued copper single crystals treated with high-density electropulses [54], as shown in Figure 1(a1,b1).

Compared with heat treated samples, the recrystallization temperature of the EPTed NiTi alloy was reduced by 200 °C, and NiTi alloy after EPT could effectively attain a homogeneous microstructure with a grain size of about 41 nm [61]. Complete recrystallization occurred after treatment by high density electropulsing (*J*_max_ = 4.8 − 5.4 × 10^3^ A∙mm^−2^) for cold-rolled TA15 (Ti-6Al-2Zr-1Mo-1V) alloy, and the degree of recrystallization increased with increasing current density [62]. The microstructure transformed from primary α lath grains to finer α equiaxed grains. Similarly, obvious recrystallization occurred in the TC4 (Ti-6Al-4V) sheet after EPT with the maximum current density *J*_max_ = 5.06 − 5.26 × 10^3^ A∙mm^−2^, and the recrystallized grains were refined to ~5 μm and uniformly distributed [63], as shown in Figure 1(a2,b2). 

The change of misorientation angle between the grains after the EPT also indicated the recrystallization during the process. The deformed microstructure was dominated by low angle grain boundaries (LAGBs), while the recrystallization grains were represented by large angle grain boundaries (HAGBs) [64]. The grains of cold-rolled AZ31 (Mg-3Al-1Zn) and AZ91 (Mg-9Al-1Zn) alloy were refined after EPT, as shown in Figure 1(a3,a4,b3,b4). Besides, the LAGBs with misorientation within 2–15° were predominant (97%), and the deformed structure with ‘necking’ appeared in the primary grain before recrystallization. After EPT, new fine recrystallized grains formed, with misorientation mainly of HAGBs larger than 15° (82%) [56,65]. 

**Figure 2:**
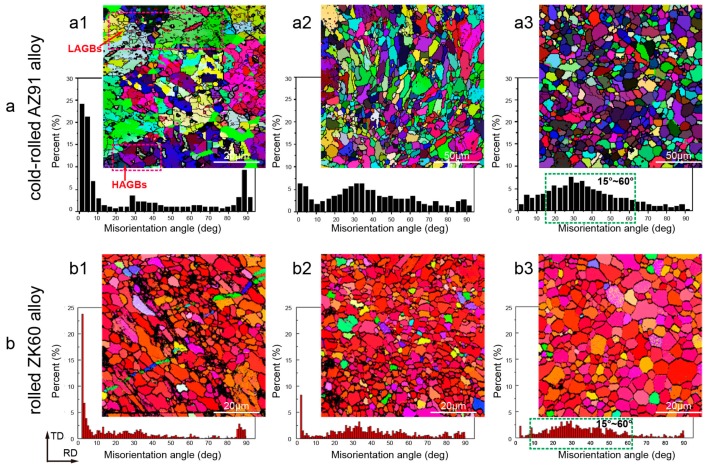
Electron back-scattered diffraction (EBSD) maps and the corresponding distributions of the misorientation angle of static recrystallization (SRX) for the magnesium alloy under EPT: (**a**) Cold-rolled AZ91 alloy with different frequencies: (**a1**) no-EPT; (**a2**) 100 Hz; and (**a3**) 110 Hz [66]. (**b**) Rolled ZK60 alloy with different pulse widths: (**b1**) 20 μs; (**b2**) 22 μs; and (**b3**) 30 μs [67].

Pulse width and frequency significantly affect the recrystallization as shown in Figure 2, and the results of EBSD revealed that with increasing frequency and pulse width, the amount of the misorientation angle distribution of 2–10° and 85–90° decreased gradually and a broad misorientation angle distribution in the range of 15–60° became predominant [66,67].

Compared to conventional heat treatment, electropulses accelerated the recrystallization of cold-rolled AZ91 alloy [68,69], and the EPT-induced crystallinity increased with the frequency and pulse width of the pulse current. The misorientation between recrystallized grains and other grains often weakened crystallographic texture until complete recrystallization. Further increase in frequency resulted in grain growth due to the higher temperatures involved and, therefore, increased crystallographic texture. The accelerated recrystallization behaviour in the AZ91 alloy under EPT is attributed to complex thermodynamic and kinetic effects in consideration of dislocation dynamics and the diffusion-controlled phase transformations.

Ma et al. [70] observed the local nanocrystallised γ-Fe grains in boron steel after EPT at a current density of 890 A∙mm^−2^. The application of electropulses reduced the thermodynamic barriers, increased nucleation rates and led to the formation of nanocrystals. In addition, accelerated spheroidization of layered eutectoid structures was observed for severely-deformed pearlitic steel (Fe-0.8C-0.2Si-0.5Mn) after EPT [57]. Spheroidization referred to the cementite flakes of nanometre size transforming into discontinuous globular grains in the pearlite structure. Processes similar to spheroidization and grain refinement in hot rolled transformation-induced plasticity (TRIP) steel (Fe-0.14C-2.1Mn-1.0Si-0.03Al-0.025Nb) and 316L stainless steel (Fe-17Cr-10Ni-3Mo-1.5Mn) after EPT have been investigated further [58,71,72], as shown in Figure 1(a5–a7,b5–b7). It was proposed that the application of high-density electropulsing was equivalent to additional free energy, leading to the formation of small globular cementite particles (about 30 nm) that were uniformly distributed in the matrix. With the increase of free energy level contributed by EPT, more interfaces would be generated for spheroidization, thus generating finer grains.

### 2.2. Phase Transitions and Grains Refinement

Previous studies have indicated that solid phase transition induced by EPT plays an important role in the formation of ultra-fine grain (UFG) structure and even nanostructure [73]. The theoretical works about the effect of electric current on phase transformation have been developed [74,75]. As a non-equilibrium process, the EPT-induced unstable solid phase in the sample at high temperature could be retained after the rapid cooling to room temperature. Electropulsing not only promotes a metastable phase to evolve towards its equilibrium state; it also enhances the stability of the metastable phase if its electrical resistivity is lower than that of stable phase [76]. As Lu et al. reported, the decomposition of stable δ-phase in 2205 stainless steel was accelerated after EPT, because the phase transition δ → γ + σ was promoted at the high temperature, which was equivalent to stabilizing the phase of γ and σ, mainly for their lower electrical resistivity than δ. 

**Figure 3:**
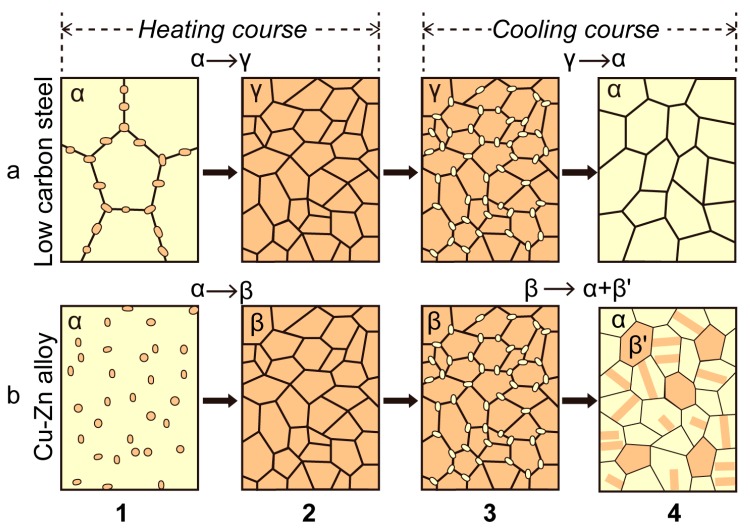
Schematic diagram of phase transition and ultra-fine grains: (**a**) low-carbon steel [77]; (**b**) Cu-Zn alloy [79]: (1) nucleation of γ or β phase, (2) grain growth of γ and β phase, (3) formation of α phase nucleation and (4) formation of ultra-fine grains and twin structure of α phase and β′ phase.

Figure 3 illustrates the four successive process of UFG structure and nanostructure formation in two-phase coarse-grained alloys by applying high density electropulses. In Figure 3a, a refined α-phase microstructure in low carbon steels (α-Fe and a small amount of pearlite) is formed after the heating/cooling cycle by EPT and will be smaller under a high cooling rate at the same heating conditions [77]. Zhou et al. found that the nano-sized γ-Fe phase could be retained after EPT, which proved the phase transition from α-Fe to γ-Fe in the course of the EPT process, for γ-Fe in the low carbon steel forms only at high temperature [78]. Zhang et al. reported that there was not only the α-Cu (Zn) phase, but also the β′-(CuZn) phase observed in EPT nanostructured H62 alloy (Cu-Zn). The phase transition in H62 alloy went through the following reaction: α-Cu (Zn) → α_1_-Cu (Zn) + β-(CuZn), as illustrated in Figure 3b. Part of the α-Cu (Zn) phase was transformed to the nano-sized α-Cu (Zn) phase. The phase transitioned from the two-phase region to the single-phase region. Some co-existing phases remained in the alloy after cooling, and the β-(CuZn) phase went through the phase transition from disordered to ordered β′-(CuZn) [79].

Ye et al. revealed that the precipitated β-Ti from Ti-6Al-4V (α + β) alloy gathered and formed continuous strips and migrated from inside worm-like particles to an equiaxed grain area after EPT in a very short duration, for which it was assumed that the nucleation rate of the phase transition α → β was accelerated [80]. Song et al. reported that fine and uniform grains with a size of 30–50 μm of as-cast TiAl alloys could be obtained from the original coarse lamellar grains of about 1000 μm by EPT. The phase transformation of γ to α took place in the α + γ dual phase field of TiAl alloys during heating. The electropulses facilitated the phase transition by decreasing thermodynamic barriers in the transition process and increased the nucleation rate [81,82]. Thus, the electropulsing effect on phase transformation can be ascribed to the promotion of nucleation rate, and the reduction of thermodynamic barrier.

The effect of dynamic EPT on phase transitions in eutectic Zn-Al-based alloys during tensile deformation was investigated by To et al. [83,84,85,86]. The results showed that electropulses significantly accelerated phase transition at two stages: (1) when ΔG < 0, the effect of EPT accelerated the supersaturated phase, decomposing into a saturated phase close to the final stable state; the phase transition reaction of α + ε → T′ + η took place when ΔG = 0; (2) when ΔG > 0, the effect of EPT increased the Gibbs free energy and facilitated the reverse process of the four phase reactions, which was T′ + η → α + ε, and also the decomposition of the η phase: ηF + αF → η′T. 

Comparing the effect of EPT on phase transition with conventional heat treatment, an additional energy term for the total Gibbs free energy results from the electric current in the conductor substantially decreased the apparent solid phase transition temperature. Jiang et al. treated aged AZ91 magnesium alloy strips by dynamic electropulses [87]. For the phase transition α + β → α′, Δ*G*_0_ denotes the energy barrier, while the free energy change of the current-carrying system of EPTΔ*G*^EPT^ can be described by:

Δ*G*^EPT^ = Δ*G*_0_ + Δ*G*_e_ (1)

where Δ*G*_e_ is an energy change due to a change in distribution of the current in the formation of a nucleus, and Δ*G*_e_ ˂ 0 according to the electrical properties of the nucleus and the matrix. It is implied that EPT substantially decreased the thermodynamic barrier and then decreased the apparent solid temperature of the β phase in the AZ91 strip. Jeong et al. observed that the precipitation of the β-Mg_17_Al_12_ phase was significantly inhibited, and even its dissolution into the α-Mg matrix was accelerated at relatively lower temperatures (~530–608 K), rather than the 673–993 K required under conventional heat treatment [88].

Recent studies suggested that EPT-induced the dissolution of secondary phases, and precipitates also contributed to the grain refinement [89]. Different sizes and densities of precipitates led to different current distributions. Qin et al. applied electropulses during the heat treatment of austenitic stainless steel (316 L) and discovered that the density of precipitates was lower and the particle size was smaller compared to the samples without treatment, as shown in Figure 1(a7,b7). The result indicated that: (i) electropulses assisted in dissolving the precipitates due to the change of the thermodynamic sequence caused by the pulse current; and (ii) the current accelerated the mass diffusion process rather than thermal effects [90].

### 2.3. Formation of Oriented Microstructure and Texture Evolution

In addition to the effects of phase transition, grain refinement and recrystallization, EPT could make some special changes in the microstructure, such as eliminating the texture formed in the previous hot working process, and generated alternative texture [91]. To a certain extent, EPT can achieve a custom structure, i.e., the direction of the long axis of the grains could be rearranged along the direction of the current [92].

From the view of electrical resistivity, Qin et al. [93] explained how the cementite sheets in ferrite-pearlite steel broke and rearranged, when treated by the electropulses. As the geometry of a phase and its structure affected the resistance of the steel, the EPT tended to construct the structure in a way that reduced the resistance. Thermodynamic calculations also showed that EPT contributed to the structural evolution of the material toward a state with lower resistivity. The tendency of lower free energy and resistivity was parallel to the current direction. From a thermodynamic point of view, the difference of free energy was the driving force for the rearrangement of cementite plates.

**Figure 4:**
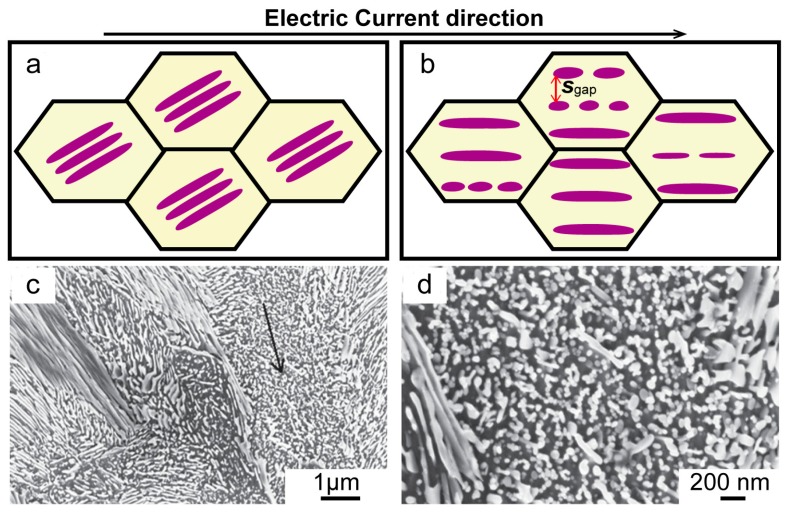
The formation of orientation microstructures in pearlite: (**a**,**b**) various configurations of pearlite microstructure [91]; (**c**,**d**) SEM images of samples after EPT [93].

In binary alloys, such as pearlitic steel containing ferrite and cementite phases, the resistivity of cementite was higher than that of ferrite. The geometrical morphology of each phase and the spatial configuration of the two phases significantly affected the total resistivity of the material. Figure 4a,b illustrated the various configurations of pearlite. The pulse current resulted in the cementite sheets being broken into discontinuous globules, which changed the geometry and spatial structure of the two phases, as shown in Figure 4b. The distance between the gap areas of the fragments affects the total resistivity according to the formula:

(2) $$R=\frac{\rho L}{S}=\frac{\rho L}{S_{gap}}$$

where ρ is the electrical resistivity, *L* is the total length of the sample, *S* is the cross-section area and *S_gap_* is the gap area between the fragmented plates, respectively. Further EPT led to the formation of newly-formed cementite plates along the current direction, thus minimizing the free energy of the system. These lamellae are almost parallel to the electric current direction, labelled with an arrow in Figure 4c. The lamellae perpendicular to the electric current direction are easier to transform into grain structures than the parallel ones [94]. The spheroidization process is controlled by the diffusion of carbon atoms from cementite to ferrite. The diffusion process is affected by physical, thermal and electrical heterogeneities.

**Figure 5:**
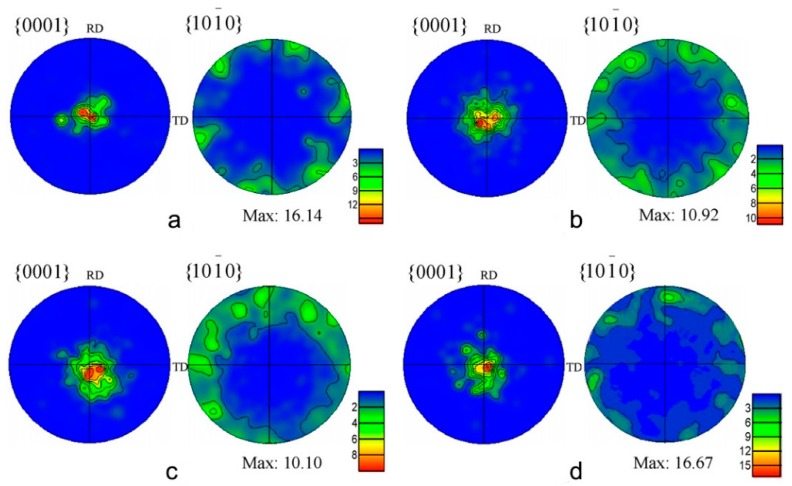
The {0001} and {10$\bar{1}0$} pole figures of the AZ91 alloy after EPT: (**a**) cold-rolled sample; (**b**) 100 Hz-EPT; (**c**) 110 Hz-EPT; (**d**) 133 Hz-EPT [68].

In the EPT copper single crystal, the arrangement of the long axis of the recrystallized grains was almost parallel to that of the initial resident slip bands formed [54]. The strength of texture {0001} of cold-rolled AZ91 alloy was very high. With the increase of frequency and inducement of recrystallization, the strength of texture decreased gradually, as shown in Figure 5 [68]. As the grains were growing, the strength of the texture increased gradually, and the grown grains were found in the same direction as the rolling direction {10$\bar{1}$0}.

## 3. Effects of EPT on the Properties of Materials

The microstructure of the material determines its mechanical and corrosion properties. After EPT, the overall mechanical properties of the material could be improved due to an optimized microstructure obtained after treatment. This review elaborates on the changes of strength, plasticity, hardness, corrosion performance and other parameters of different materials after EPT, as well as the parameters that affect these properties.

### 3.1. The EPT-Induced Microstructure-Properties Relationship of Materials 

#### 3.1.1. Titanium Alloy

As a widely-used, but hard-to-deform industrial material, titanium and its alloys are suitable for electropulsing treatment, as an alternative to hot deforming treatments. Tskhondiya et al. [95] showed that EPT could be combined with traditional deformation treatment, such as rolling, and achieved better performance than single treatment. The decrease of flow stress under the action of pulse current could give rise to a softening effect that influences the elongation of material [96,97]. Table 1 lists the mechanical properties of pure titanium and several titanium alloys before and after EPT. The strength of cold-rolled sheets of commercial TA1-A CP Ti treated by high density electropulsing was significantly higher than that of the conventionally-annealed samples. Song et al. analysed the results and suggested that electropulsing induced the formation of fine equal-axed grains and a lamellar microstructure, which divided the grains to reduce the effective slip distance and to raise the flow stress via the Hall–Petch mechanism. Thus, the strength was higher for EPTed CP Ti sheets compared to conventional annealing ones, while retaining the high ductility [98]. Song et al. also compared the effect of EPT and annealing on the TC4 titanium alloy sheets and demonstrated significantly enhanced plastic deformation behaviour in a very short time by EPT [63], as the tensile elongation was increased by 48.6%, while the yield strength was decreased by 19.8%. Besides the recrystallization and restrained grain growth due to the low energy and very short duration of high density electropulsing, the decreased dislocation density attributed to the ‘drifting electrons’ also contributed to the enhanced formality. A significantly increased total elongation (93%) by EPT was found in the cold-rolled TA15 sheet, which was induced by the damage healing and local recrystallization [62]. 

**Figure 6:**
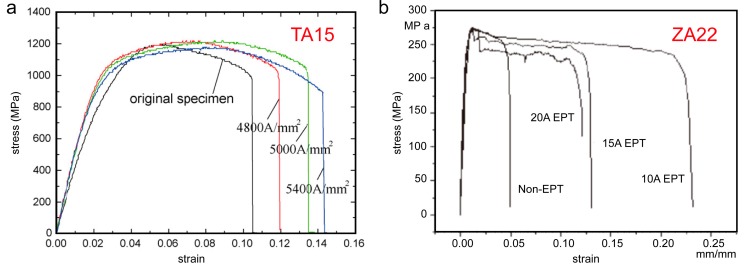
Stress-strain curves of TA15 alloys and ZA22 alloys at different pulse current densities: (**a**) TA15 [62]; and (**b**) ZA22 [86].

The anisotropy behaviour of materials during deformation may contribute to the grains’ orientation hardening near the basal [99,100]. The current density of EPT plays an important role in the mechanical properties anisotropy evolution of materials. The influence of the current density on the elongation is shown in Figure 6a. There is anisotropy in the TA15 sheets electropulsed with maximum current density of 4.8 × 10^3^ A⋅mm^−2^ and 5.0 × 10^3^ A⋅mm^−2^. Under high density pulse current, the differences of mechanical properties almost diminished at a maximum current density of 5.4 × 10^3^ A·mm^−2^ [62]. Similar results were found in the Ti-6Al-4V alloy and showed that EPT weakened the anisotropy behaviour of materials during plastic deformation, which may be attracted to the formation of non-directional fine equiaxed grains; the selective effect of current also supplies the directional energy input for grains’ evolution to relieve mechanical properties’ anisotropy [101].

#### 3.1.2. Zinc Alloy

The ‘electro-plastic effect’ in metallic materials was first discovered during the directional deformation of a zinc single crystal. It has been found that EPT could reduce the brittleness and improve the plasticity of zinc and its alloys. Table 2 lists the mechanical performance of the zinc alloys before and after EPT. Increases in elongation were achieved by applying pulse electric current to the deformed ZA22 alloy at room temperature [86]. As shown in Figure 6b, the elongation of ZA22 alloy increased by 437% under the optimum current (10 A EPT). The authors claimed that electropulsing effectively accelerated the movement of dislocation, which significantly improved the plasticity of the alloy. The electropulses homogenized the distribution of dislocations. That is, electropulses induced the T’ phase precipitates and changed the dislocation pinning [102]. Thus, even at higher deformation rates (e.g., work hardening rate or shrinkage rate), the inelastic strain and plastic elongation could be improved.

#### 3.1.3. Magnesium Alloy

Magnesium and its alloys have been described as being promising in industrial and biomedical fields for their high specific strength and low mass density, but the low strength and poor room temperature formability of magnesium alloys have limited their application. It is the hexagonal close-packed (hcp) structure of pure magnesium and most of its alloys that implies the poor plasticity at room temperature; thus, elevated temperature during process or subsequent conventional heat treatment is essential to improve their forming and, finally, the mechanical properties. Since the discovery of electropulse-induced grain refinement on metals without an excessive increase in processing temperature, it has played an important role in improving the strength and plasticity of magnesium alloys by increasing grain boundary slip, according to the Hall–Petch relationship [103]. Table 3 lists the mechanical properties of the magnesium alloys before and after EPT. 

**Figure 7:**
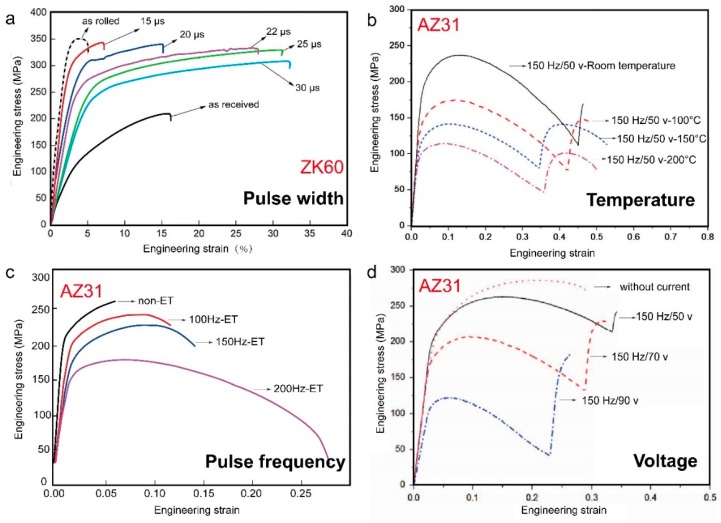
Stress-strain curves for magnesium alloys: (**a**) pulse width [67]; (**b**) temperature [89]; (**c**) pulse frequency [13]; and (**d**) voltage [114].

The softening degree of deformed ZK60 magnesium alloy treated by EPT was found higher than the equivalent heat treatment at the same processing time by Jin et al.; the hardness was decreased and the elongation increased, with the tensile stress of sheets improved; details can be seen in Table 3. The influence of work hardening was eliminated because of much finer recrystallization grains obtained due to static recrystallization [104]. Chu et al. showed that the elongation to failure of AZ31 alloy by EPT at room temperature was improved due to the electroplastic effect [105]. The electropulse-induced inverse eutectic reaction (α + β = L) occurred at the necking fracture zone due to an elevated transient temperature. The presence of appropriate amount of inverse eutectic liquid phases could optimise the deformation mechanism and improve the ductility of the materials, while the ductility deteriorated as a result of the over-reaction of the inverse eutectic process [106]. The combined treatments of aging, cold-rolling and EPT were carried out on the AZ91 alloy strips. The ultimate tensile strength, yield strength and elongation to failure of the EPT-samples were improved by 11–12%, 10% and 70–75%, respectively [87]. Jiang et al. have noted that the hardness of the cold-rolled AZ91 magnesium alloy decreased with the increased pulse frequency, which was attributed to a reduction in dislocation density [52,68]. Figure 7 shows that the EPT pulse width, processing temperature, pulse frequency and voltage significantly affected the tensile properties of magnesium alloys.

In the complex stress state, which is more relevant to industrial sheet processing, such as rolling, the dominant deformation mechanism of magnesium alloys is twinning [107]. Kuang et al. [108,109,110] studied the effect of pulsed electric current on the twinning behaviour of AZ31. The ‘current-induced’ twins, including improved activities of {10$\bar{1}$1} contraction twins (CT) and {10$\bar{1}$1}–{10$\bar{1}$2} double twins (DT), existed in rare earth (RE)-free AZ31 alloy and played similar roles during rolling in AZ31 as those ‘RE-induced’ twins in the RE-containing alloy [111]; while the {10$\bar{1}$2} extension twins (ET) were found insensitive to the current and can be activated in electroplastic-differential speed rolling, contributing to the stored energy relieving at the grain boundaries and facilitating dynamic recrystallization (DRX) [107]. Additionally, the grains grew along the stretching direction after EPT, and the twins changed the original direction of the grains and released the stress concentration. Meanwhile, an ‘electronic wind effect’ contributed to the movement of the dislocations by disrupting their entanglement. These effects caused by electropulses activated more slip systems, which in turn improved the plasticity with an acceptable decrease of tensile strength.

#### 3.1.4. Iron and Steel

Table 4 lists the mechanical performances of the steel samples before and after EPT. Zhou et al. found that the tensile strength, elongation and the microhardness of low-carbon steel (0.07% C) were significantly improved after EPT due to the formation of equiaxed ferrite grains of 0.5–3 μm. The fracture mode of the EPT steel samples changed from brittle to ductile due to the presence of microcrystals and nanocrystals [77]. Samuel et al. [57] investigated the influence of the magnitude of electric current pulses on the severely-deformed pearlitic steel. Their results showed a decrease in Vickers hardness in all of the EPT samples. The decrease may arise from the different degrees of refinement and spheroidization of pearlite and cementite particles within their microstructures. Rahnama et al. [58] studied the relationship between hardness, yield strength, tensile strength and layer spacing of EPTed ferro-pearlitic duplex steel (0.14% C). The results suggested that it followed the Hall–Petch relationship, which confirmed the results of Samuel’s experiments. It was found that the softening effect in EPTed steels may be ascribed to the following: (i) the increased pitch of the lamellae and the subsequent spheroidization, which in turn allow dislocations to have enough space to move freely within the matrix; (ii) voids being removed, thereby forming a precipitate-free zone (PFZ) along the grain boundary. 

Lu et al. found that the mechanical properties of dual phase steel (DP600) were significantly improved by EPT-tempering [115]. The tensile strength increased by 38.98%, yield strength by 8.90% and hardness by 20.9%, presumably due to the formation of nano-cementite particles in the ultra-fine ferrite grains. As a result, the softening problem of dual phase steel was effectively delayed and even repaired. Thus, EPT was a suitable alternative to delay or even to repair the temper softening problem, in comparison to other non-isothermal tempering methods.

**Figure 8:**
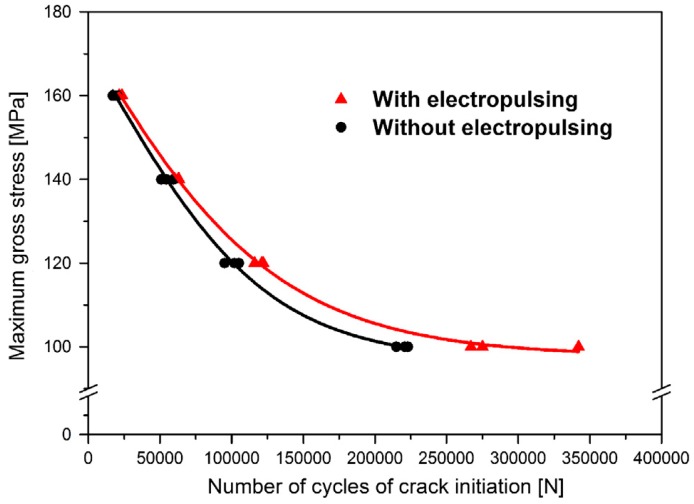
*S-N* curves of stainless steel (SUS316) before and after EPT [116].

Tang et al. [116] showed that the hardness decreased and fatigue lifetime increased for EPTed SUS316 stainless steel, and the effect of electric current on *S-N* curve is shown in Figure 8; the results indicated that the fatigue crack initiation was delayed by the application of electric current, which is related to the healing of the fatigue damage, including the recovery of the residual plastic strain and the strain hardening. G. Lesiuk et al. [117] demonstrated a similar effect in AISI 304 steel (0.04% C, 1.1% Mn, 0.41% Si, 0.0437% P, 0.0044% S, 18.16% Cr, 8% Ni, 0.0335% Mo, 0.1% V, 0.32% Cu), and the differences in fatigue lifetimes are much higher with low stress amplitudes than with high stress level.

### 3.2. Effect of EPT on the Corrosion Performance

It has been reported that the EPT can affect the corrosion performance of metals and alloys. Long et al. [118] studied the effect of EPT on improving the corrosion resistance of cold brushed steel. The high density EPT can effectively eliminate the internal residual stress and reduce the density of dislocation, therefore reducing the stress corrosion rate. Xiao et al. [119] studied the effect of high density EPT on the corrosion behaviour of the X70 pipeline steel. The results showed that high density EPT led to the grain refinement of X70 pipeline steel and improved the resistance to local corrosion. Wang et al. [120] compared the effect of high energy EPT and conventional heat treatment on the corrosion resistance of AZ31 magnesium alloy strips. The high energy EPT and conventional heat treatment both improved the corrosion resistance of the as-received and non-heat treated AZ31 strip in 3.5% NaCl solution. They assumed that this was related to the recrystallization and the reduction of the dislocation density after the treatments. However, more corrosive microcells may form due to the formation of finer grains after EPT, which may result in the decrease of corrosion resistance. Despite the grain refinement, Qin et al. analysed the enhancement of corrosion resistance in steel. The results suggested that the increase of the total fraction of Σ3 boundaries by electropulsing provided excellent corrosion resistance and lower electrical resistivity than that of a normal grain boundary [121,122].

## 4. Application of EPT in Crack Healing

The EPT-induced rapid and localized heating tends to give rise to heterogeneous thermal expansion and thus the development of thermal stress. The peak stress *σ*_max_ can be estimated by temperature rise following the linear function:

σ_max_ ≈ *E*αΔT (3)

Here, *E* is Young’s modulus, *α* is the thermal expansion coefficient and ΔT is the temperature rise. ΔT related to the current density can be calculated by Equation (1). With the increase of integral current density, the thermal stress can give rise to plastic deformation during EPT processing, both of which will affect the crack propagation and healing [123].

**Figure 9:**
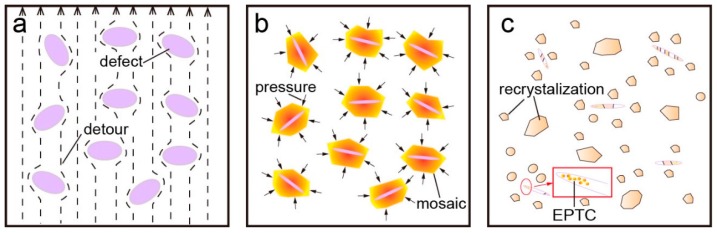
Schematic diagram of defect healing and recrystallization: (**a**) plastic deformation to form defects and defective areas of the current bypass; (**b**) the formation of pressure around the metal defects; (**c**) healing defects, recrystallization and high energy EPT cladding (EPTC) [55,124].

Damage and fatigue will lead to the development, growth and accumulation of microcracks during the service life of materials. The electropulses have been shown to slow down, prevent or even heal the cracks in metals, thereby increasing the service life of materials. Ye et al. [124] and Song et al. [55] compared the crack morphology of the TC4 pre-deformed plate before and after EPT. They found that the crack healed from the tip, and the width of cracks in the middle decreased, involving that recrystallization of equiaxed grains in front of the crack tip, and they bridged the crack, thereby indicating that EPT could prevent microcrack growth and increase the deformation limit. The above-mentioned crack healing observations were used to explain the healing process, as illustrated in Figure 9. 

**Figure 10:**
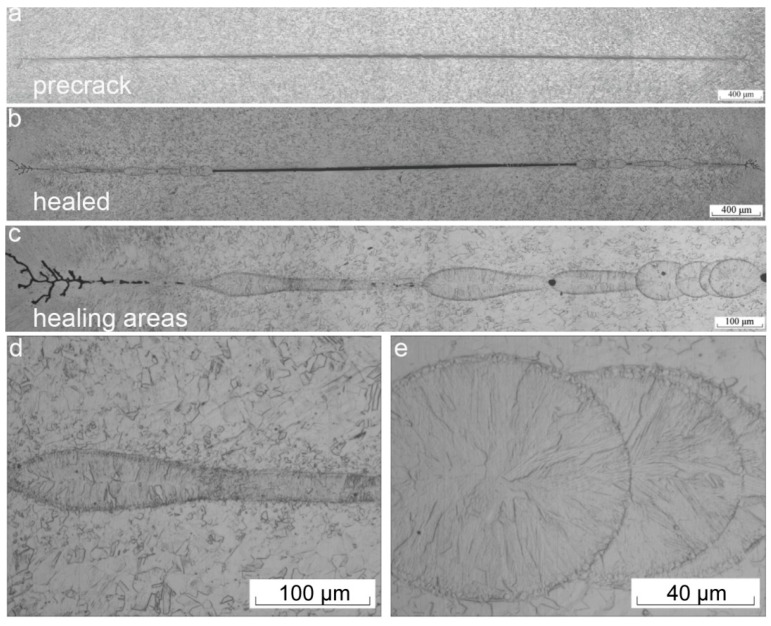
Morphology of healed cracks and healing areas: (**a**) pre-crack; (**b**) healed area; (**c**) healing areas; (**d**,**e**) partially enlarged view [125].

With the increase of the pulse numbers, the whole crack can be closed. Yu et al. [125] used multiple electric pulses to heal the cracks in SUS304 stainless steel. After seven times of electropulsing treatment, continuous healing on both extremities of a crack was observed until complete healing was attained, as shown in Figure 10. It was suggested that the detour and joule heating effects were the prime reasons for the crack healing. The process of rapid melting and solidification occurred in the healing areas. At the same time, columnar crystals and a fine recrystallization zone formed. The healing effect generated by each pulse discharge was not consistent, so that there was a tendency for diminished microstructural changes. Three mechanisms of this reaction process were proposed from the perspectives of dislocation movement, atomic diffusion and thermo-compressive stress. 

**Figure 11:**
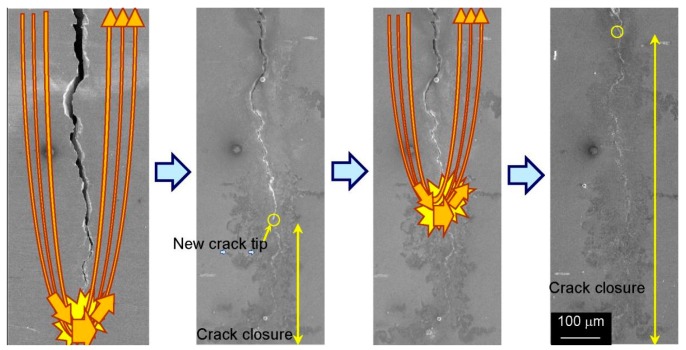
Closure of an entire fatigue crack by multiple applications of high-density electropulsing [128].

Further study on the crack healing effect in medium carbon steel suggested that the crack was electrically stressed to produce a low-temperature plasma. On the other hand, there was atomic movement towards the crack face, and this motion would be influenced by the local thermal expansion in the vicinity of the crack [126]. The two crack faces merged to achieve crack healing when the thermal expansion was sufficient. In addition, the original microstructure in the area without crack remains unchanged. Thus, the principles of physics during EPT will cause preferential interaction with the defective microstructure during the crack healing process. The healing of fatigue crack by high-density electropulsing in austenitic stainless steel was studied by Hosoi et al. [127,128]. The selective effect was found during crack healing. For the current flowing along the crack because of the electric resistance being higher on the crack surface, the thermal compressive stress was generated along the crack to heal the tip, resulting in the crack tip transferring in the direction of the notch. The position of the current concentration continuously transferred in the direction of the notch. Figure 11 shows the whole process.

Conventional heat treatment requires prolonged heating at high temperatures, promote atomic diffusion and healing damage, which inevitably results in coarse grains. However, there was no abnormal grain growth observed after EPT. In this aspect, EPT is superior to conventional annealing. Qiao et al. [129] studied the crack healing process in aluminium alloys. For damaged 4043 aluminium alloy specimens, the strengths of EPT samples (0.5-s electric pulses) were close to those of the undamaged samples. For the fatigue-damaged 2024 aluminium alloy specimens, the fatigue life was significantly prolonged by 0.8 s of EPT. Observations on the microstructures revealed that the cracks in the samples after EPT healed and recrystallization occurred.

## 5. Theoretical Discussions on the EPT

The influence of pulse current on the structure and properties of materials has been extensively explored, but there are still arguments about the microscopic mechanism behind it. In a single pulse, the energy, heat and stress are instantaneously input into the material, leading to microscopic changes in structure and, therefore, the properties and performance of the materials. In addition, the EPT effects may vary and depend on the specific type of material being processed. This review summarized the persuasive discussions that have been covered in the literature up to date.

### 5.1. Thermal and Athermal Effects

At present, the dominant view of the grain refinement and recrystallization due to the EPT is that the pulse current increases the nucleation rate and decreases the growth rate of the recrystallized nuclei. Many researchers attempted to explain this procedure, and the thermal and athermal effects have been taken into account. The thermal effect was joule heating, while the athermal effect was attributed to the ‘electronic wind’ on atom diffusion. 

**Figure 12:**
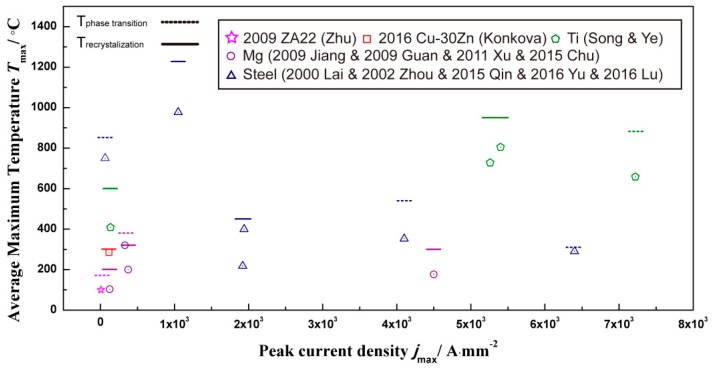
The average maximum temperature of different metals and alloys with different peak current densities [24,30,52,53,59,63,67,78,82,85,98,101,112,114,123,125,131].

The temperature rising during EPT due to joule heating plays an important role in the evolution of the microstructure. When the current flows through materials, a high thermal energy comes from electron motion transported to the materials [130] and caused the temperature rise, which can be estimated theoretically as follows [57]:

ΔT = ρj^2^t (C_p_d)^−1^ (4)

where j is the peak current density, t is pulse time and ρ, C_p_ and d are the electrical resistivity, the specific heat capacity and density, respectively. Because the heated duration between each electropulse is about several microseconds, much shorter than the interval time, the cumulative effect of temperature rise could be neglected [6]. Figure 12 describes the relationship of peak current density and the average maximum temperature change (T_max_ = T_0_ + ΔT, T_0_ is the initial temperature) by electropulsing, which can be calculated by Equation (4). In general, the calculated value of ΔT mainly depends on the current density and the material itself; T_max_ of different materials are lower than the required recrystallization and phase transition temperature (Figure 12). This phenomenon denotes that joule heating is not the only effect during EPT, which makes the difference in the evolution of the microstructure from the conventional heating treatment.

According to Zhu et al., the root mean square (RMS) current density mainly led to the increase of temperature in NiTi alloy [132]. Contrary to the traditional heat treatment, the enhanced nucleation rate in EPT is because grain nuclei can be formed at any location with different distortion degrees for more joule heat produced by the structure with low distortion degree [61].

Guan et al. [65] concluded that the thermal and athermal effects are the main reasons for the enhancement of nucleation rate at low temperature recrystallization in AZ31 magnesium alloy during EPT. This combination of factors was thought to provide the additional driving force for enhancing grain boundary migration, as a result of the accelerated interchange of vacancies and atoms through the increased driving pressure provided by electropulsing. Accelerated boundary migration then enhanced the early stage nucleation and retardation of subsequent grain growth, which in turn gives rise to complete recrystallization within a few seconds. Liu et al. [56] analysed the mechanism of grain refinement in AZ31 magnesium under EPT and claimed that in order to obtain refined recrystallization grains, the athermal effect should be strengthened, while the thermal effect should be suppressed as the subsequent growth of the recrystallization grains was driven by the Joule heating rather than the athermal effect. However, in EPTed AZ91 alloy, Jiang et al. found that the recrystallization rate only increased when the thermal effect due to Joule heating was sufficiently large to accelerate dislocation and sub-crystal growth. The influence of the athermal effect on these factors was too small to induce recrystallization behaviour alone [69]. By comparing the true stress-true strain curves of AZ31B alloy under uniaxial tensile tests, the athermal effect under electropulses was confirmed [133,134,135].

Research on the recrystallisation behaviour in EPT cold-rolled silicon steel strips was carried out by Hu et al. [136]. They discussed a similar theory of electron wind accelerated dislocation climb, which led to an enhanced nucleation rate and retarded growth of recrystallized grain. “Electron migration” was thought to be an important factor in the electropulse-induced low temperature recrystallization process; this atomic flux was attributed to the thermal and athermal effects. Additionally, the dislocation climb to the sub-grain boundary was closely related to the total flux of the diffusion atoms induced by athermal effects. To prove the existence of the coupling of thermal and athermal effect during the whole EPT process in processing of Ti-6Al-4V, Ye et al. recorded the temperature evolution in the heating and cooling period [137].

### 5.2. Accumulation and Annihilation Effects

The occurrence of recrystallization depends on the recovery of dislocations and atomic diffusion. It was reported that the number of dislocations per unit time (*n_c_*) moving into the sub-grain boundaries is expressed as follows:

(5) $$\frac{dn}{dt}=\frac{j\Omega }{b}$$

where *j* is the total atom diffusing flux, consists of that due to thermal effect and that owing to athermal effect, corresponding to the vacancy, *b* the Burgers vector and Ω the atom volume. According to Equation (5), movement and annihilation of dislocation speed up at the sub-grain boundaries, as the *j* increases markedly in EPT [86]. In the EPT of Al-Li alloy, Liu et al. observed that pulsed current promoted the dislocation slip and atom diffusion, accelerated the dislocation to enter the grain boundary and increased the sub-crystal angle [138]. 

Xu et al. also claimed that the dynamic recrystallization in magnesium alloys was caused by a change in dislocation density near the grain boundary. Under the EPT, the effect of electron wind promoted the accumulation of dislocations, leading to coarse grain boundaries. The coupling of heat and electron migration effects eliminated dislocations, which caused the rearrangement of dislocations. This means that the action of the electric pulse is composed of two parts, the accumulation effect caused by the electron wind and the annihilation effect caused by the coupling of heat and electric transport effect [53]. Therefore, the reason in terms of dislocation motion why current pulses accelerate the dynamic recrystallization process is thought to be due to the combined effects of accumulation and annihilation.

Xiao et al. [139] argued that the annihilation of dislocations caused by high density current pulses increased the gradient of dislocation density, over local inhomogeneous dislocation structures. When the dislocation density gradient accumulated to a certain extent, the low dislocation density region became the recrystallization nuclei, and the high density dislocation region formed a recrystallized grain boundary. Song et al. [140] applied the dynamic mechanical analyser to compare the internal friction and modulus of the cold-rolled pure titanium plate samples under EPT with the normal annealed samples and demonstrated that the EPT decreased tangling of dislocations and enhanced dislocation mobility. 

**Figure 13:**
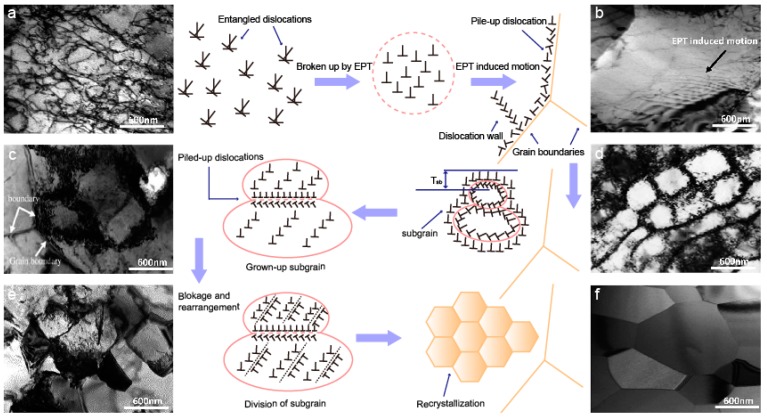
Schematic diagram of dislocation behaviour during recovery and recrystallization and (**a**–**f**) corresponding TEM images of the Ti-6Al-4V alloy during the process [124].

Ye et al. [124] studied the dislocation behaviour induced by EPT in Ti-6Al-4V for complete recrystallization. Figure 13 illustrated the mechanism and the corresponding TEM images of EPT for the recovery and complete recrystallization process, from the perspective of dislocation behaviour, including nucleation and growth of dislocation cells or subgrains and the formation of recrystallization. Large numbers of entangled dislocations were observed in titanium alloy samples after cold deformation, which blocked the further motion of dislocations, as depicted in Figure 13a. The rapidly broken up tangled dislocations and the subsequent dislocation migration and rearrangement were induced by the EPT athermal effect, as shown in Figure 13b. Thus, dislocations piled up at grain boundaries, and dislocation walls tended to form in this period, as shown in Figure 13c. Increasing the voltage of EPT induced the strengthening effect of the EPT thermal effect, which is another important factor in accelerating the nucleation of sub-grains with a thick cell wall near the grain boundaries. Further microstructure evolutions induced by the coupling of thermal and athermal effects were presented as follows: (i) growth of subgrains, (ii) reducing the thickness of the subgrain boundary and (iii) sub-division of the subgrains. Complete recrystallization was finally acquired and presented in Figure 13f [124].

## 6. Challenges and Future Outlook

### 6.1. The Microstructure and Properties

It has been proven that high density EPT is an effective approach to improve the microstructure and the overall mechanical properties of metals. The improvement of the mechanical properties of the material is manifested by the enhancement of strength, plasticity and toughness mainly due to the strengthening effect of fine grains. There are areas of ultra-fine grain structure and nanostructure coexisting in the majority EPTed metals due to the rapid heating and cooling process. The future outlook demands such features as oriented texture to be dominant and manufactured in a controlled fashion rather than a curious and scientifically interesting anomaly [141]. The most appropriate pre-treatment before EPT is cold work, such as rolling and extrusion, since the effect of electric current pulses on recrystallization can be amplified in the pre-deformed materials [142,143]. The art in the EPT processing is to adjust the electropulsing parameters so that only partial recrystallization arises in order to maintain nanostructured features.

The EPT methods showed considerable promise in decreasing the cost of grain refining treatments, improving the forming limit of the material and mending defects such as microcracks. However, there are associated challenges with regard to attaining uniform grain refinement structure. It is foreseen that specific developments, in alloy modification, surface engineering and plastic processing will be primary drivers to grow EPT.

The mechanical properties of EPTed alloys may be tailored by adjusting the microstructure. Some strategies include: (i) varying the parameters of different materials with regard to pulse current, such as current density, pulse width and frequency, to achieve the desired microstructure; and (ii) using other manufacturing techniques, such as traditional heat treatment to modify the as-received materials. Since complicated effects occurred in a single process, such as the thermal and athermal effects and the accumulation and annihilation effects, which explained the mechanism of EPT processing from the thermodynamics and dynamics point of views, further research is still required to fully understand and clarify the mechanisms underlying the electropulsing process. 

However, the technique is known to sometimes cause undesirable effects on metals, which is not mentioned by most researchers in this field, but cannot be neglected. For example, the EPT-induced local transient heating led to the thermal expansion and subsequent cooling. The residual tensile stress caused by EPT could lead to crack initiation and propagation, which is undesirable on metals.

### 6.2. Applications in the Future

EPT involves unique processing parameters. Thus, there are broad applications of EPT for improving other properties of materials left unexplored. For instance, defects of the material can be recovered without knowing the exact position of the defect since the pulse current will self-select these regions. Further growth markets will be uncovered because of the new materials that are coming to market and are being tailored microstructurally via EPT. Wu et al. [144] described an approach that formed nanocrystalline cores embedded in amorphous glassy shells of a magnesium alloy. This alloy and associated EPT treatment combined the strengthening benefits of nanocrystallinity with those of amorphization. This dual-phase material exhibited near-ideal strength at room temperature without sample size effects. Thus, EPT may refine the internal grains in the amorphous material to improve the strength and corrosion resistance of the material. 

There is still no perfect theory and model that can be used for the effects of EPT; the studies up to date can only partially explain it, so future research of the mechanisms by which the microstructures are transformed by the high density electrical pulses with short duration and a more comprehensive review of the effects of electrical pulses on the metallic alloys will be meaningful research directions.

Electropulse technology is a proven method to beneficially modify the local microstructure of metals. The physics and metallurgical principles of EPT are fertile grounds for knowledge and discovery. There are no known industrial applications for EPT up to date. However, electropulse-assisted forming is a promising method for improving the mechanical property of materials [145,146,147]. In addition, in the field of surface engineering, the EPT can be used to improve the mechanical properties and microstructure of the surface coatings [148,149,150,151,152,153,154]. The successful scientific and academic studies foreshadow positive manufacturing outcomes.

## Figures and Tables

**Table 1:** Mechanical properties of pure titanium and titanium alloys before and after EPT.

Sample State		Ultimate Tensile Strength (MPa)	Yield Strength (MPa)	Tensile Elongation (%)	Ref.
TA15 sheet	cold-rolled	1175	-	7.2	[62]
	electropulsed	1100	-	13.9	
	increase by (%)	−6	-	+93	
TC4 sheet	annealed	1033	936	15.6	[63]
	electropulsed	947	750	23.18	
	increase by (%)	−8.3	−19.8	+48.6	
TA1-A CP-Ti sheet	annealed	300	210	40	[98]
	electropulsed	400	300	31.5	
	increase by (%)	+33	+43	−21	

**Table 2:** Mechanical properties of zinc alloys before and after EPT.

Sample State		Peak Current Density (A/mm^2^)	Ultimate Tensile Strength (MPa)	Tensile Elongation (%)	Ref.
ZA22 sheet	non-EPT	-	298	4.5	[86]
	EPT	8.13	272	6.4	
		12.32	300	8.5	
		15.75	260 *	5.5	
		21.21	275	6.4	

* The mechanical properties are estimated from Figure 1 in [86].

**Table 3:** Mechanical properties of magnesium alloys before and after EPT.

Sample State		Hardness (HV)	Ultimate Tensile Strength (MPa)	Yield Strength (MPa)	Tensile Elongation (%)	Refs.
ZK60 sheet	cold rolled	848	210	—	15.7	[67,104]
	Electropulsed	728	320	—	30	
	increase by%	−14.15	+52.4	—	+91	
AZ91 strip	cold rolled	—	330	245	16.2	[87]
	Electropulsed	—	362~370	270	27.6~28.4	
	increase by%	—	+11~12	+10	+70~75	
AZ31 strip	cold rolled	—	315 *	280 *	10	[112,113]
	Electropulsed	—	295~262 *	265~185 *	29~43	
	increase by%	—	−6~18	−5~34 *	+190~330	

* The mechanical properties are estimated from Figure 4 in [112].

**Table 4:** Mechanical properties of steels before and after EPT. TRIP, transformation-induced plasticity.

Sample State		Ultimate Tensile Strength (MPa)	Yield Strength (MPa)	Tensile Elongation (%)	Vickers Hardness (HV)	Ref.
TRIP sheet	hot rolled	700 *	570 *	23 *	230 *	[58]
	electropulsed	630 *	480 *	26 *	180 *	
	increase by (%)	−0	−15.79	+13.04	−21.74	
Low-carbon steel sheet	annealed	580	—	40	179	[77]
	electropulsed	1040	—	45	325	
	increase by (%)	+79	—	+13	+82	
DP600 sheet	cold rolled	1034.25	773.26	3.31	301 ± 8	[115]
	electropulsed	1126.33	1074.66	3.12	364 ± 12	
	increase by (%)	+8.90	+38.98	−5.74	+20.9	

* The mechanical properties are estimated rom Figure 8, Figure 9, Figure 10 and Figure 11 in [58].
